# Upregulation of SIX4 indicates poor clinical outcome and promotes tumor growth and cell metastasis in esophageal squamous cell carcinoma

**DOI:** 10.1111/1759-7714.13832

**Published:** 2021-01-22

**Authors:** Yanping Li, Xiaomei Jiang, Xiaoyan Yan, Yanzheng Wang

**Affiliations:** ^1^ Department of Gastroenterology Rizhao Hospital of TCM Rizhao China; ^2^ Outpatient Department Qingdao Eighth People's Hospital Qingdao China; ^3^ Health Management Department Qingdao Eighth People's Hospital Qingdao China; ^4^ Department of Clinical Laboratory Yantaishan Hospital Yantai China

**Keywords:** Cell metastasis, esophageal squamous cell carcinoma, SIX4, tumor growth

## Abstract

**Background:**

The role of sine oculis homeobox 4 (SIX4) has been found in some malignant tumors. However, there have been few studies on the function of SIX4 in esophageal squamous cell carcinoma (ESCC). This study aimed to explore the regulatory mechanism of SIX4 in ESCC.

**Methods:**

RT‐qPCR and Western blot analysis were used to measure mRNA and protein expression. The function of SIX4 was investigated using CCK‐8, colony formation, flow cytometry, wound healing and transwell assays. A mouse xenograft tumor assay was designed to perform in vivo experiments.

**Results:**

SIX4 was upregulated in ESCC and indicated poor clinical outcomes in ESCC patients. Functionally, knockdown of SIX4 inhibited cell proliferation and induced apoptosis in ESCC. In addition, the silencing of SIX4 inhibited cell migration, invasion and EMT in ESCC. More importantly, upregulation of SIX4 could activate the PI3K/AKT pathway in ESCC cells and promote tumor growth in vivo.

**Conclusions:**

Upregulation of SIX4 indicates poor clinical outcomes in ESCC patients and promotes tumor growth and cell metastasis in ESCC.

## Graphical abstract

1. SIX4 was upregulated in ESCC and indicated poor clinical outcomes in ESCC patients. 2. SIX4 promotes tumor growth and cell metastasis in ESCC. 3. Upregulation of SIX4 could activate the PI3K/AKT pathway in ESCC cells.

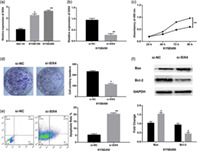

## INTRODUCTION

Esophageal cancer is a relatively common tumor of the digestive tract. It is a malignant tumor that occurs in the epithelial tissue of the esophagus, accounting for 2% of all malignant tumors.^1^ It is estimated that about 200 000 people die of esophageal cancer each year worldwide, and it is one of the most common malignant tumors that is extremely harmful to peoples' lives and health.^2^ The main subtype of esophageal cancer is esophageal squamous cell carcinoma (ESCC). The main risk factors for ESCC are drinking and smoking, but other diseases that cause chronic irritation of the esophageal mucosa may also increase the risk of ESCC.^3^ After radical radiotherapy and radical surgery, it has been previously reported that the five‐year survival rate of early ESCC can reach more than 90%,^4^ and after surgery, radiotherapy, and chemotherapy, the five‐year survival rate of advanced ESCC can reach about 30%–40%.^5^ Although the diagnosis and treatment of ESCC has been improved, the prognosis of ESCC patients is not optimistic. Therefore, it is necessary for us to explore novel therapeutic targets for ESCC.

The sine oculis homeobox (SIX) protein family has been reported to play an important role in the development of vertebrate tissues and organs.^6^ SIX has been found to have transcriptional dysregulation or post‐translational modification in primary tumors and metastatic lesions.^7^ The SIX family includes six members (SIX1–SIX6), of which the role of SIX1 in the occurrence and development of cancer has been widely investigated.^8^ However, the role of SIX4 in tumorigenesis remains largely unknown. SIX4 has been reported to promote metastasis through STAT3 activation in breast cancer.^9^ In addition, miR‐621 was found to inhibit the malignant progression of non‐small cell lung cancer (NSCLC) by targeting SIX4, indicating that SIX4 functions as an oncogene in NSCLC.^10^ However, the role and regulatory mechanisms of SIX4 in ESCC have not yet been elucidated.

The epithelial‐mesenchymal transition (EMT) process has become an important mechanism for the distant metastasis of epithelial malignant tumors, including ESCC.^11^ EMT is usually characterized by downregulation of epithelial molecules (E‐cadherin) and upregulation of mesenchymal markers (N‐cadherin, vimentin). These cells have the ability to reconstruct distant metastatic colonies.^12^ PI3K/AKT signaling pathway is involved in the regulation of multiple cell functions such as proliferation, differentiation, apoptosis and glucose transport.^13^ The increase of PI3K activity is often associated with the tumorigenesis of cancers. In addition, AKT can phosphorylate target proteins to exert antiapoptotic effects. It has been reported that AKT phosphorylates the Bcl‐2 family member BAD, which prevents it from binding to Bcl‐XL to induce apoptosis.^14^ In addition, AKT can inhibit the activity of the proteolytic enzyme caspase‐9 and prevent the activation of the apoptotic cascade.^15^ However, the effect of SIX4 on EMT and PI3K/AKT signaling pathway in ESCC has not been reported in previous studies.

Therefore, the abnormal expression and function of SIX4 was investigated in ESCC. At the same time, the effect of SIX4 on EMT and PI3K/AKT signaling pathway in ESCC was also elucidated.

## METHODS

### Bioinformatic analysis

GEPIA (http://gepia.cancer-pku.cn/) is a newly developed interactive web server for analyzing RNA sequencing expression data from the 9736 tumor samples and 8587 normal samples of The Cancer Genome Atlas (TCGA). The screening conditions were: (i) datasets selection: esophageal carcinoma (ESCA); (ii) gene: SIX4; and (iii) expression DIY: boxplot, Stage plot.

### Clinical tissue samples

The ESCC tissues and matched paracarcinoma tissues were obtained from 86 patients after the approval of the Ethics Committee of Yantaishan Hospital. Each participant only received surgery and signed the written informed consents prior to this study. All samples were snap‐frozen in liquid nitrogen and then stored at −80°C.

### Cell culture

Normal esophageal cell line (Het‐1A) and ESCC cell lines (KYSE150, KYSE450) were purchased from the type Culture Collection of Chinese Academy of Sciences (Shanghai, China). BEBM medium (Het‐1A), Ham's F12 medium (KYSE150) or RPMI‐1640 (KYSE450) medium supplemented with 10% fetal bovine serum (FBS) was used to incubate the cells at 37°C with 5% CO_2_.

### Cell transfection

The SIX4‐specific siRNA (si‐SIX4) was used to downregulate SIX4 expression, and a nonsilencing siRNA (si‐NC) oligonucleotide was used as a negative control (GenePharma). The cDNA encoding SIX4 was subcloned into the vector pcDNA3.1 (pcDNA‐SIX4, Invitrogen). The empty pcDNA3.1 vector (pcDNA‐NC) was used as a control. Next, they were transfected into KYSE450 cells using Lipofectamine 2000 (Invitrogen), respectively.

### Quantitative real‐time PCR

The isolation of total RNA was performed using TRIZOL reagent (Invitrogen). SIX4 was reverse transcribed by using PrimeScript RT Master Mix (Takara). RT‐qPCR assay was performed using real‐time PCR mixture assays (Takara) based on the manufacturer's instructions. The relative quantification of SIX4 was performed using the 2^‐△△Ct^ method, with GAPDH used as an internal control.

### Western blot analysis

Protein samples were lysed using RIPA buffer (Beyotime). Next, proteins were separated using 10% SDS‐PAGE and blocked with 5% nonfat milk. Protein samples were then transferred to PVDF membranes. The protein sample was incubated with Bax (rabbit polyclonal antibody; dilution, 1:500; catalog no. 137321), Bcl‐2 (rabbit polyclonal; dilution, 1:500; catalog no. ab45171), E‐cadherin (rabbit monoclonal; dilution, 1:1000; catalog no. ab1416; Abcam), N‐cadherin (rabbit polyclonal; dilution, 1:1000; catalog no. ab18203; Abcam), vimentin (rabbit monoclonal; dilution, 1:1000; catalog no. ab217673; Abcam), SIX4 (rabbit polyclonal; dilution, 1:1000; catalog no. ab176713) and GAPDH (rabbit monoclonal, dilution, 1:1000; catalog no. ab181602) primary antibodies (Abcam) overnight at 4°C. After washing, the protein samples were incubated with secondary antibodies (mouse anti‐rabbit, dilution, 1:2000; catalog no. 58802; Abcam) for 1 h. Finally, protein bands were assessed using the ECL kit (Beyotime).

### Cell viability assay

Transfected KYSE450 cells were incubated for 24, 48, 72 and 96 h at 37°C in a humidified atmosphere with 5% CO_2_, respectively. Cell counting kit‐8 (CCK‐8) (Dojindo) was then used to incubate the cells for 4 h. The absorbance at 450 nm was observed using a microplate reader (Molecular Devices).

### Colony formation assay

The transfected cells were seeded into six‐well plates (400 cells/well) and incubated for 7–14 days. The culture was terminated after the colonies could be seen by the naked eye. Next, the cells were fixed in methanol and stained with 0.1% crystal purple staining solution for 30 min. Finally, colony imaging was counted.

### Flow cytometry

The transfected cells were digested by ethylenediaminetetraacetic acid (EDTA)‐free trypsin and collected in a centrifugal tube. Next, the cells were mixed with 5 μl Annexin V‐FITC and PI. The cells were then incubated at room temperature for 5 min in the dark. Finally, a flow cytometer was used to detect cell apoptosis.

### Wound healing assay

The transfected KYSE450 cells (2 × 10^3^ cells/well) prepared on a glass petri dish were incubated overnight at 37°C to allow the cells to adhere to the plate. A straight scratch was then made with a plastic pipette tip after cell samples reached a confluence of 80%. The cells were then rinsed in PBS to remove the separated cells. Finally, the wound was imaged at 0 and 24 h using a light microscope (Olympus).

### Cell invasion and migration assay

Cell migration or invasion was measured in the upper chamber without or with Matrigel. Transfected KYSE450 cells were added to the upper chamber and DMEM medium supplemented with 10% fetal bovine serum (FBS) was added to the lower chamber. After 24 h, the moving cells were stained with 0.1% crystal violet. The cells were calculated and visualized under light microscopy.

### Mouse xenograft tumor assay

Ten 6‐week‐old male SCID mice were obtained from the National Laboratory Animal Center (Beijing, China). This study was approved by the Institutional Animal Care and Use Committee of Yantaishan Hospital. The KYSE150 cells containing pcDNA‐NC or pcDNA‐SIX4 were injected into the right flank of the mice. Next, tumor volume was monitored every four days. The tumor volume was then calculated by length × width^2^/2. After four weeks, the nude mice were sacrificed, and the tumors were collected and weighed.

### Statistical analysis

Data are shown as mean ± SD. Experimental data were analyzed using Student's *t*‐test and one‐way ANOVA in SPSS 17.0 or Graphpad Prism 6 software. *p*‐values < 0.05 were considered statistically significant.

## RESULTS

### Upregulation of SIX4 is found in ESCC tissues

**FIGURE 1:**
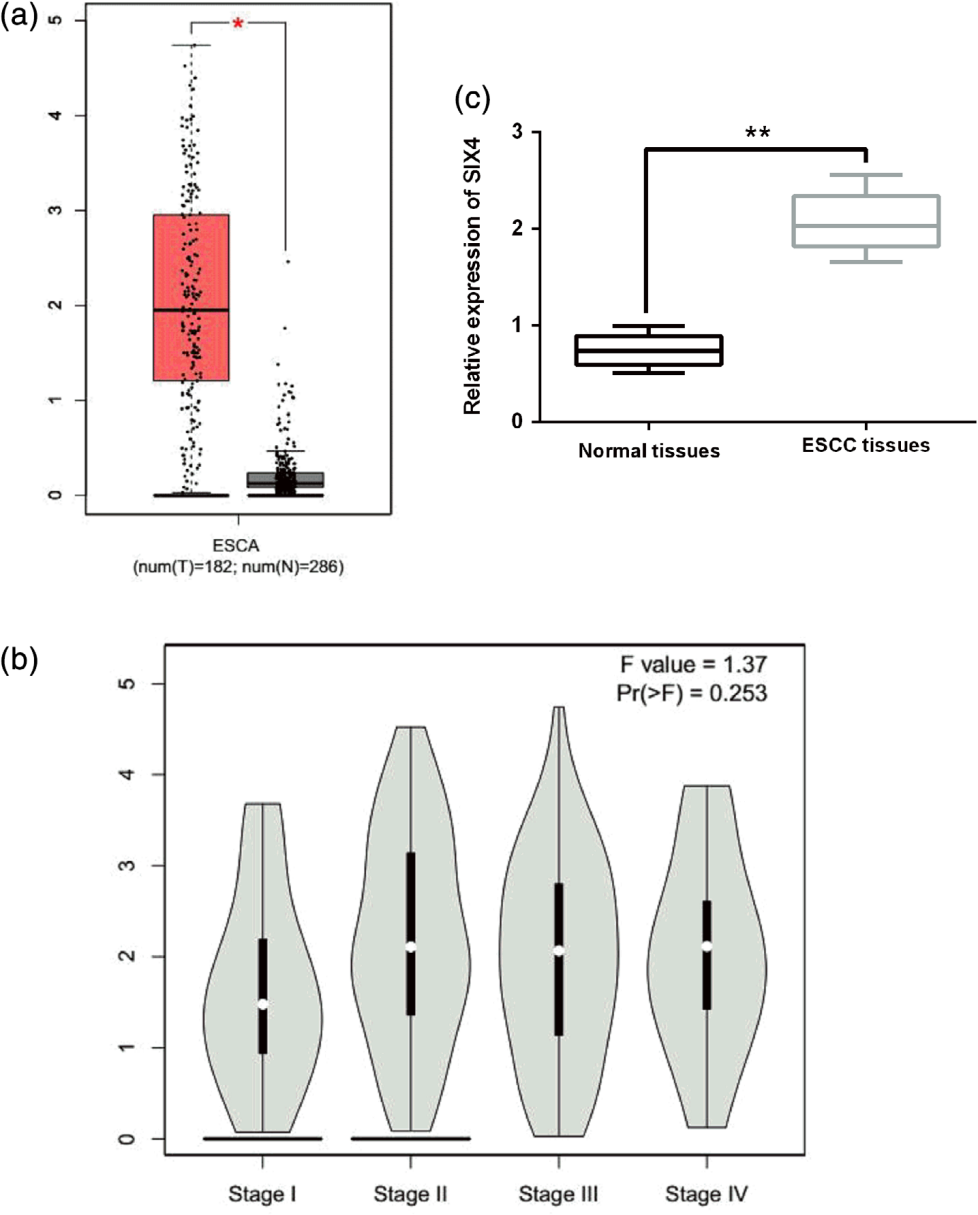
Upregulation of SIX4 is found in ESCC tissues. (a) Expression level of SIX4 in ESCC tissues (*n* = 182) and normal tissues (*n* = 286) analyzed in TGCA database. (b) Expression level of SIX4 in ESCC tissues at stage I, II, III and IV analyzed in TGCA database. (c) SIX4 expression was detected in ESCC tissues and normal tissues (*n* = 86) by RT‐qPCR. *p < 0.05, **p < 0.01

First, the GEPIA database showed that SIX4 was upregulated in ESCA tissues compared to normal tissues (*p* < 0.05, Figure 1(a)). In addition, the GEPIA database also indicated that SIX4 expression was increased in ESCA patients at stage II, III and IV compared to that in stage I. However, the difference was not significant (*p* > 0.05, Figure 1(b)). In this study, we also found that SIX4 expression was increased in ESCC tissues compared to normal tissues (*p* < 0.01, Figure 1(c)). The relationship between abnormal expressions of SIX4 and clinical features in ESCC patients was analyzed. As shown in Table 1, SIX4 expression was associated with lymph node metastasis in ESCC patients (*p* < 0.05, Table 1). These results indicated that SIX4 may be involved in the pathogenesis of ESCC.

**TABLE 1:** Relationship between SIX4 expression and the clinicopathological characteristics in 86 ESCC patients

Characteristics	Number of cases (*n* = 86)	SIX4		*p*‐value
		High	Low	
**Age (years)**				0.34
≥ 60	52 (60.5%)	36 (41.9%)	16 (18.6%)	
<60	34 (39.5%)	22 (25.5%)	12 (14.0%)	
**Gender**				0.67
Male	49 (57.0%)	32 (37.2%)	17 (19.8%)	
Female	37 (43.0%)	26 (30.2%)	11 (12.8%)	
**Tumor size (cm)**				0.16
≥4	39 (45.3%)	21 (24.4%)	18 (20.9%)	
<4	47 (54.7%)	37 (43.0%)	10 (11.7%)	
**Differentiation**				0.31
Well and moderately	60 (69.8%)	40 (46.5%)	20 (23.3%)	
Poorly	26 (30.2%)	18 (20.9%)	8 (9.30%)	
**TNM stage**				0.06
I + II	56 (65.0%)	43 (50.0%)	13 (15.0%)	
III + IV	30 (35.0%)	15 (17.5%)	15 (17.5%)	
**Lymph node metastasis**				
Positive	58 (67.4%)	42 (48.8%)	16 (18.6%)	0.02^a^
Negative	28 (32.6%)	16 (18.6%)	12 (14.0%)	

*Note*: Statistical analyses were performed by the χ^2^ test.

Abbreviation: TNM, tumor‐node‐metastasis.

^a^ *p* < 0.05 was considered significant.

### Knockdown of SIX4 inhibits cell proliferation and induces apoptosis in ESCC

**FIGURE 2:**
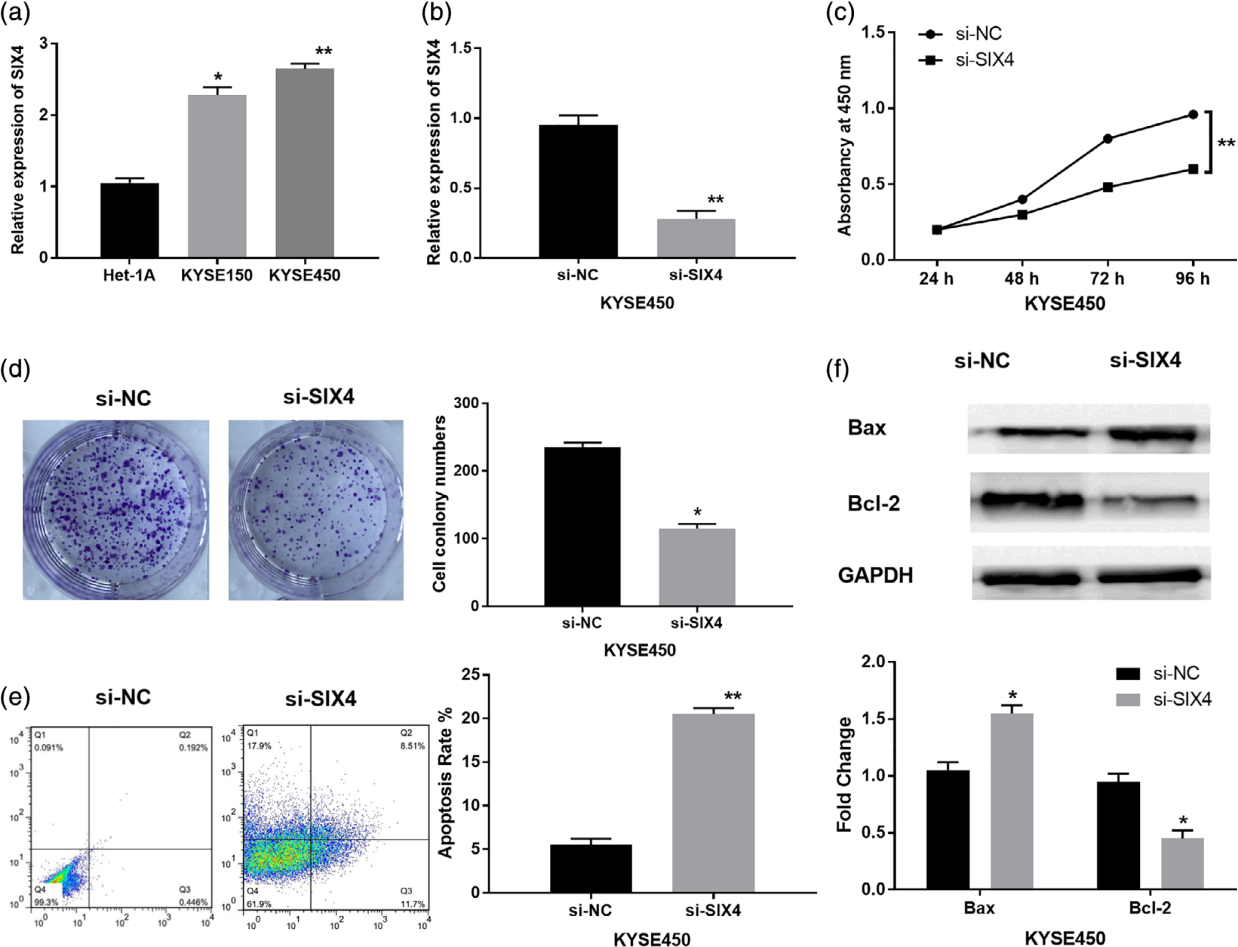
Knockdown of SIX4 inhibits cell proliferation and induces apoptosis in ESCC. (a) SIX4 expression in ESCC cell lines KYSE150 and KYSE450 and normal esophageal cell line (Het‐1A). (b) SIX4 expression in KYSE450 cells with si‐SIX4 or si‐NC. (C) Cell proliferation was detected in KYSE450 cells with si‐SIX4 or si‐NC by CCK‐8 assay. (d, e) Cell clone number and apoptosis percentage were detected in KYSE450 cells with si‐SIX4 or si‐NC. (f) Apoptosis‐related proteins (Bcl‐2/Bax) was detected in KYSE450 cells with si‐SIX4 or si‐NC by Western blot analysis. *p < 0.05, **p < 0.01

Next, the expression level of SIX4 was detected in ESCC cells. Compared with normal esophageal cells Het‐1A, upregulation of SIX4 was identified in ESCC cell lines KYSE150 and KYSE450 (*p* < 0.01, Figure 2(a)). KYSE450 cells were used to explore the role of SIX4 in ESCC due to its significant difference in SIX4 expression. Then, si‐SIX4 or si‐NC was transfected into KYSE450 ESCC cells. RT‐qPCR showed that SIX4 expression was significantly reduced by si‐SIX4 in KYSE450 cells (*p* < 0.01, Figure 2(b)). Functionally, knockdown of SIX4 was found to inhibit cell proliferation in KYSE450 cells (*p* < 0.01, Figure 2(c)). The colony formation assay showed that had considerably cell colonies in si‐SIX4 group was fewer than that of si‐NC group (*p* < 0.05, Figure 2(d)). At the same time, downregulation of SIX4 induced apoptosis in KYSE450 cells (*p* < 0.01, Figure 2(e)). Next, the effect of SIX4 on apoptosis‐related proteins (Bcl‐2/Bax) was detected in KYSE450 cells. We found that si‐SIX4 promoted Bax expression and decreased the survival gene Bcl‐2 expression (*p* < 0.05, Figure 2(f)). All these results indicated that SIX4 could promote cell proliferation and inhibit apoptosis in ESCC.

### Knockdown of SIX4 inhibits cell metastasis and EMT in ESCC

**FIGURE 3:**
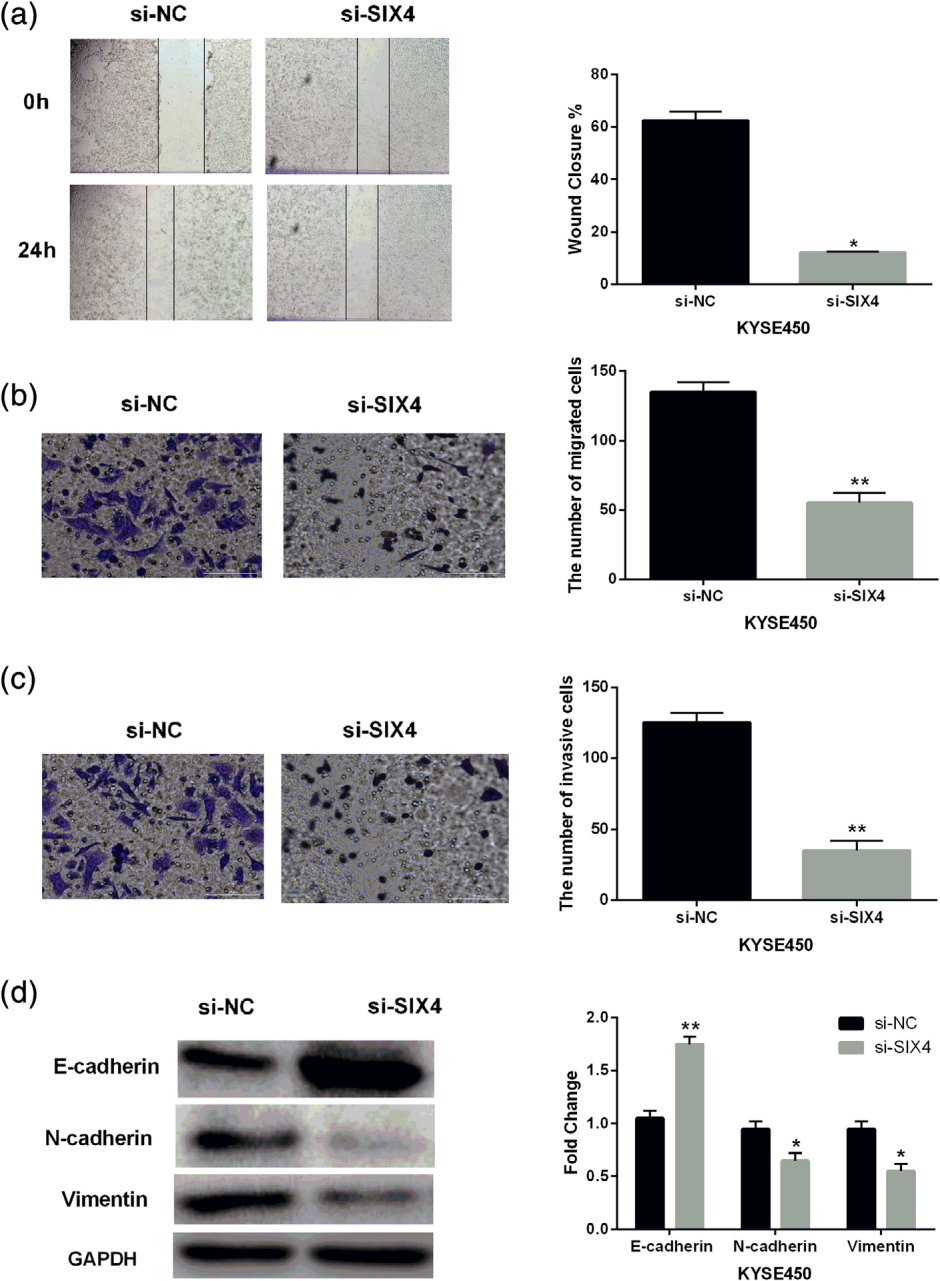
Knockdown of SIX4 inhibits cell metastasis and EMT in ESCC. (a) Cell migration was detected in KYSE450 cells with si‐SIX4 or si‐NC by wound healing assay. (b, c) Cell migration and invasion were detected in KYSE450 cells with si‐SIX4 or si‐NC by transwell assay. (d) E‐cadherin, N‐cadherin and vimentin expression was detected in KYSE450 cells with si‐SIX4 or si‐NC by Western blot analysis. *p < 0.05, **p < 0.01

Next, the effect of SIX4 on cell metastasis was investigated in ESCC. Wound healing assay showed that silencing of SIX4 significantly decreased the velocity of cell movements. The percentage of wound closure at 24 h was decreased from 62.3% in the si‐NC group to 12.1% in the si‐SIX4 group (*p* < 0.05, Figure 3(a)). In addition, transwell assay showed that knockdown of SIX4 restrained cell migration and invasion in KYSE450 cells (*p* < 0.01, Figure 3(b), (c)). In order to explain the mechanism underlying mobility of SIX4, the expression of EMT components were detected in KYSE450 cells. The results showed that knockdown of SIX4 reduced N‐cadherin and vimentin expression and increased E‐cadherin expression (Figure 3(d)). These findings imply that silencing of SIX4 inhibits cell metastasis and EMT in ESCC.

### Upregulation of SIX4 promotes ESCC tumor growth in vivo

**FIGURE 4:**
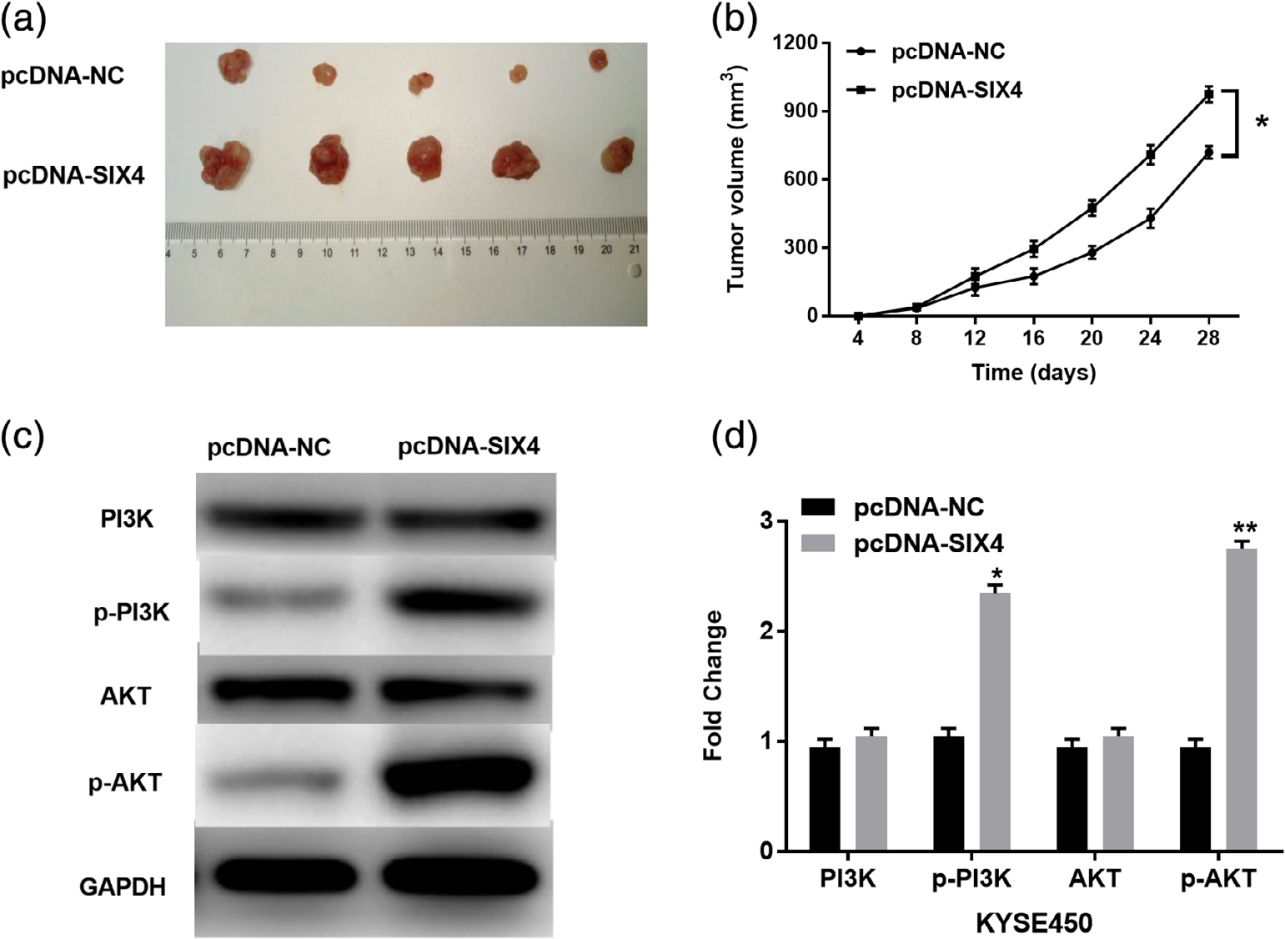
Upregulation of SIX4 promotes ESCC tumor growth in vivo. (a, b) The tumor volume and growth curve in nude mice after being injected with KYSE450 cells expressing pcDNA‐NC or pcDNA‐SIX4. (c, d) PI3K, AKT, p‐PI3K and p‐AKT expression was detected in KYSE450 cells with pcDNA‐NC or pcDNA‐SIX4 by Western blot analysis. *p < 0.05, **p < 0.01

Finally, KYSE450 cells with SIX4 vector were subcutaneously injected into nude mice. Compared with the control group, the tumor volume was increased in mice with SIX4 vector (Figure 4(a)). Tumor growth in SIX4 vector group was also faster than that of the control group (*p* < 0.01, Figure 4(b)). In addition, the effect of SIX4 on PI3K/AKT pathway was investigated in KYSE450 cells. Western blot analysis showed that upregulation of SIX4 promoted p‐PI3K and p‐AKT expressions in KYSE450 cells (*p* < 0.01, Figure 4(c), (d)). However, PI3K and AKT expressions were not affected by SIX4 in KYSE450 cells. These results indicated that upregulation of SIX4 could activate the PI3K/AKT pathway in ESCC cells and promote tumor growth in vivo.

## DISCUSSION

Recently, the important roles of SIX4 have been found in various physiological processes. For example, Six4 can regulate male sex determination and mouse gonadal development.^16^ Mice doubly deficient in homeobox genes Six4 and Six5 showed ventral body wall defects as those seen in human omphalocele.^17^ In this study, SIX4 was upregulated in ESCC tissues and cells. Upregulation of SIX4 was associated with cell differentiation and lymph node metastasis in ESCC patients. Functionally, knockdown of SIX4 inhibited cell proliferation and induced apoptosis in ESCC. In addition, the silencing of SIX4 inhibited cell migration and invasion in ESCC. These results imply that SIX4 serves as a tumor promoter in the progression of ESCC.

Consistent with our results, SIX4 expression has been reported to be increased in ESCC. Abnormal expression of SIX4 in ESCC was correlated with clinical prognosis.^18^ In addition, SIX4 has been found to act as a master regulator of oncogenes that promotes tumorigenesis in NSCLC cells.^19^ Wu et al. found that knockdown of SIX4 inhibited cell growth, invasion, and the EMT of glioblastoma.^20^ Moreover, it has been reported that downregulation of SIX4 led to bladder cancer cell cycle arrest and apoptosis.^21^ These findings are similar to our results. In this study, we also found that upregulation of SIX4 could promote EMT and PI3K/AKT pathway in ESCC cells.

As we all know, EMT is an important process in the distance metastasis of cancer cells.^22^ Here, we found that knockdown of SIX4 reduced N‐cadherin and vimentin expression and increased E‐cadherin expression, indicating that SIX4 promotes EMT in ESCC. Consistent with our results, SIX4 has been proposed to promote metastasis in breast cancer by activating EMT.^9^ In addition, SIX4 was found to activate AKT and promote tumor angiogenesis in solid tumor progression.^23^ Li et al. also reported that SIX4 promoted cell metastasis via activation of the PI3K‐AKT pathway in colorectal cancer.^24^ In this study, we also found that upregulation of SIX4 could activate the PI3K/AKT pathway in ESCC by promoting p‐PI3K and p‐AKT expressions. More importantly, upregulation of SIX4 was found to promote ESCC tumor growth in vivo in this study, which has not been reported in previous studies. All these findings indicate that upregulation of SIX4 can promote the tumorigenesis of ESCC by activating EMT and PI3K/AKT pathway.

In conclusion, for the first time, this study revealed that SIX4 is upregulated in ESCC and indicates poor clinical outcomes in ESCC patients. Functionally, downregulation of SIX4 inhibits cell proliferation and metastasis in ESCC. Moreover, upregulation of SIX4 can promote ESCC tumor growth in vivo. This study will extend the understanding of SIX4 role in ESCC tumorigenesis.

## CONFLICT OF INTEREST

The authors declare that they have no competing interests.
